# Safety and efficacy of directly‐acting antiviral therapy for chronic hepatitis C virus in elderly people

**DOI:** 10.1002/agm2.12190

**Published:** 2021-12-21

**Authors:** Adriano De Santis, Daniela Maggi, Federica Lubrano Lobianco

**Affiliations:** ^1^ Department of Translational and Precision Medicine Sapienza University Rome Italy

**Keywords:** DAAs, efficacy, elderly, HCV, safety

## Abstract

**Introduction:**

In Italy, the prevalence of hepatitis C virus (HCV) infection is higher in the elderly, although the efficacy and safety of treatment in this population has not been extensively studied. Moreover, little is known about how much pharmacological interaction affects eligibility to treatment and to what extent the treatment affects subsequent outcomes.

**Methods:**

We retrospectively analyzed the efficacy and safety of directly acting antivirals (DAAs), drug‐to‐drug interactions, and post‐treatment outcomes in 138 patients with HCV aged 70 years or older, who were consecutively treated in our center between 2015 and 2020.

**Results:**

The mean age was 77 years old (range = 70–95 years old). The Cumulative Illness Rating Scale of pretherapy severity was classified as moderate to severe in 65% of patients. Fifty‐five patients (40%) presented compensated cirrhosis, eight of which were complicated by hepatocellular carcinoma (HCC) and all were cured before treatment. One hundred two patients (74%) were taking two or more drugs (range = 0–5 concomitant drugs registered) and in 29 patients (21%) we found potential drug‐to‐drug interaction. In 11 of those 29 patients (38%), we were forced to change the chronic therapy, when all therapeutic regimens were equal in terms of efficacy and interactions, to avoid potentially serious drug interactions. One serious adverse event occurred in our sample population (i.e., diverticular bleeding due to interaction with direct oral anticoagulants [DOACs]), whereas mild side effects occurred in 37% of patients. The undetectability of HCV RNA at the end of treatment was achieved in 97% of patients, whereas a sustained virological response (SVR) 12 and SVR 24 were obtained in 98% of patients. When comparing pretherapy with post‐therapy data, after a medium follow‐up of 15 months (median = 1 year, minimum = 2 months, and maximum = 4 years), we observed a reduction in the incidence of episodes of liver decompensation in patients with cirrhosis and a slight increase in the incidence of HCC (with 6 recurrent and 5 de novo HCC), diagnosed within 13 months from the end of therapy. In all patients, we found a significant improvement in all ultrasound variables and a significant reduction in the elastographic measurements. No significant differences in outcomes were observed dividing the population into patients aged ≥ 80 and < 80 years old.

**Conclusions:**

Directly acting antiviral therapy was found to be safe and effective in elderly people, and, despite the large number of concomitant drugs, pharmacological interactions appeared to not affect the adherence to therapy or the incidence of adverse events. Side effects were mostly independent from the type of DAA used and from the burden of comorbidity. In long‐term follow‐up, the benefit of DAA therapy mainly concerned liver pathology and should be strongly advised in patients with cirrhosis. The therapy was found to not affect extrahepatic comorbidities but allowed to end follow‐up in noncirrhotic patients with savings in terms of resources. Finally, patients should not be excluded based on age if they have a good performance status.

## Graphical abstract

We retrospectively analyzed data of 138 HCV patients aged 70 years or older, who were consecutively treated in our center between 2015 and 2020. We found out that in a real‐life population, HCV treatment is safe (with only a few potential drug‐to‐drug interactions that require management and few adverse events), effective (98% SVR at 12 weeks), and useful (it reduce decompensating events in cirrhotics and ameliorates prognostics score and clinical parameters).

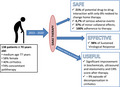

## 1 INTRODUCTION

The mean age of patients with chronic hepatitis C virus (HCV) infection and the number of elderly patients with advanced liver disease is gradually increasing in many countries, including Italy.^1^, ^2^, ^3^, ^4^, ^5^, ^6^ Many screening strategies have been implemented, although they always concern high‐risk groups, excluding the elderly population from the analysis. This risk strategy is unsuccessful in the perspective of a global eradication of the disease because patients aged 70 years or more are the main HCV pool and can only increase over time, due to the cohort effect. Furthermore, these patients are also the main users of the National Health Service and require frequent and intensive care, becoming a potential vector of infection for their caregivers.

Many studies have also shown that the age in itself and the age of onset of HCV infection significantly impact the degree of fibrosis: older individuals have an increased risk of progressing to cirrhosis and of developing complications.^7^, ^8^, ^9^, ^10^, ^11^, ^12^, ^13^, ^14^

Older patients have always been considered difficult to treat and not suitable for interferon therapies.^15^, ^16^, ^17^, ^18^ However, the second generation of directly acting antiviral drugs (DAAs) and the expansion of the prescribing criteria, have made all patients with a known infection virtually suitable for antiviral treatments. Some studies have shown that DAAs are highly effective, safe, and tolerable even in elderly patients. Most of them are based on extrapolated data from large cohorts of phase III studies and the few real‐life studies have been limited to analyzing the efficacy and safety without providing follow‐up data.^19^, ^20^, ^21^, ^22^, ^23^, ^24^, ^25^, ^26^, ^27^, ^28^, ^29^

Many clinicians do not begin DAA treatment in the elderly, as they are concerned of the many concomitant drugs and their possible interactions. Furthermore, it is still unclear which is the impact that antiviral therapy has on extrahepatic outcomes and the long‐term benefit of viral eradication in this population needs further studies. Few cost‐utility studies have been specifically applied to treating hepatitis C in the elderly,^30^ and, because of comorbidities and a shorter life expectancy, the cost‐effectiveness ratio increases with age making the therapy less attractive for elderly patients. For all these reasons, these patients have often been neglected by avoiding treatment, although the current guidelines do not consider age as an element of choice in starting treatment.^31^, ^32^

The elderly, in the perspective of a global eradication of the disease, should be considered as a real special population to screen and treat like other subjects considered at high risk. Searching for more evidence in this area is fundamental for the correct allocation of health resources and health equality.

This study aims to evaluate not only the efficacy and safety of DAA treatment on a real‐life population of patients with HCV but also to assess the impact of drug‐to‐drug interactions on the eligibility for treatment and on the natural history of liver disease. Furthermore, we analyzed the trend of hepatic and extrahepatic variables following treatment to define the overall outcome of elderly patients.

## 2 MATERIALS AND METHODS

In this retrospective study, we selected patients aged 70 years or older at the time of the start of treatment from a cohort of 575 outpatients treated with new‐generation direct antivirals from 2015 to 2020 at the Division of Gastroenterology, Policlinico Umberto I, Rome (Italy).

### 2.1 Inclusion criteria

- Patients ≥ 70 years of age with chronic HCV infection eligible for DAA therapy. Eligibility for treatment was assessed according to current clinical practices, assessing the general health conditions, potential drug interactions, and the patient’s willingness to undergo treatment. Only an 85‐year‐old patient in poor general condition and poor prognosis was excluded from access to treatment.

### 2.2 Exclusion criteria

- Patients < 70 years old at the beginning of treatment.

From an overall cohort of 575 patients with chronic HCV infection, we selected 138 patients who were over 70 years old at the start of treatment (24%). We collected the following data before and after DAA therapy:

- Personal and anamnestic data, in particular, age, gender, body mass index (BMI), comorbidities, concomitant drug therapy, and Cumulative Illness Rating Scale (CIRS).^33^
- Parameters related to liver disease, such as the presence of cirrhosis, duration of illness, and episodes of decompensation.
- Ultrasound and laboratory parameters.
- Parameters related to DAA therapy, such as duration, drug interactions^34^ and side effects.
- Cause of death, if deceased during therapy or follow‐up.

The pretherapy blood biochemical data was compared with the data collected 6 months after the end of therapy. If the data at 6 months was not available, we considered the data collected 3 months after the end of therapy.

Each patient underwent an ultrasound scan before and after therapy. The pre‐therapy ultrasound data was compared with the data of the last ultrasound in our possession after the end of the therapy (if comparable quantitative measures were reported). Elastometry with Acoustic Radiation Force Impulse (ARFI) or Fibroscan was evaluated before and after therapy, comparing ARFI with ARFI and Fibroscan with Fibroscan. The medium time from the end of therapy and the ultrasound/elastometry was 409 days. Comparable ultrasound scans were available for 111 patients (81%) and elastometry for 105 patients (76%, in 66 evaluated with ARFI and in 39 with Fibroscan).

The clinical conditions, concomitant drugs, and comorbidities were extrapolated from the last visit before starting therapy and after the end of antiviral therapy, covering a medium follow‐up of 15 months (median = 1 year, minimum = 2 months, and maximum = 4 years). From these data, we retrospectively calculated the CIRS score and data on mortality.

The research was conducted in accordance with the principles embodied in the Declaration of Helsinki and in accordance with local statutory requirements. All participants provided written informed consent to collect their clinical data anonymously. This work has been approved by the Research Commission and the Academic Senate of the La Sapienza University of Rome (registration number AR11916B89455B46).

### 2.3 Statistical analysis

The data collected were analyzed with NCSS2020 software. Quantitative variables were described in terms of mean, standard deviation, and range, whereas qualitative variables were expressed as frequencies and percentages. Comparisons of numerical variables were performed with the *t* test for paired data, whereas qualitative variables with the chi‐square test. A logistic regression analysis was performed to determine if there were any variables associated with the onset of side effects. A *P* value below 0.05 was considered significant.

## 3 RESULTS

### 3.1 Baseline characteristics of patients

The baseline characteristics of the patients of our sample population are summarized in Table 1. Our dataset was mostly composed of women (58%) with a median age of 77 years old (70–95 years old) and illness duration of 17 years on average (1–34 years). The main risk factor for HCV infection was the anamnestic presence of surgical or dental procedures without blood transfusions (45.6%). In most cases, there were no concomitant causes of liver damage (75.4%). When present, the main concomitant causes of liver damage were alcohol (10%) and steatosis (9.4%). The most represented genotypes of our dataset were 1B (56.5%) and 2 (36.2%). Ninety‐eight patients (71%) were naïve to any treatment, whereas 40 patients had already been exposed to previous therapies, of which 38 were to an interferon‐based therapy and two to a DAA treatment (who were both relapsers).

**TABLE 1:** General characteristics of patients of our dataset

Gender, F	80/138		58%
Age, y	77.36 ± 4.97 (70–95)		
BMI	25.19 ± 3.91 (16–37.8)		
Genotype	1A	5/138	3.6%
	1B	78/138	56.5%
	2	50/138	36.2%
	3	2/138	1.4%
	4	1/138	0.7%
	Mixed	2/138	1.4%
Illness duration	17.26 ± 8.78 (1–34)		
Contributing causes of liver damage	HBV	2/138	1.4%
	Alcol	14/138	10%
	Steatosis	13/138	9.4%
	Autoimmune	1/138	0.7%
	Hemochromatosis	4/138	2.9%
	None	104/138	75.4%
Risk factors for HCV	Surgery/dentistry	63/138	45.6%
	Blood transfusions	15/138	10.9%
	IDA	1/138	0.7%
	Infected family member	13/138	9.4%
	Accidental infection at work	1/138	0.7%
	Not known	43/138	32.6%
CIRS of pretherapy severity	2	48/138	34.7%
	3	42/138	30.4%
	4	48/138	34.7%
Comorbidity index	0	45/138	32.6%
	1	70/138	50.7%
	2	16/138	11.6%
	3	4/138	2.9%
	4	1/138	0.72%
	5	2/138	1.4%
Concomitant drugs	None	13/138	9.4%
	Monotherapy	23/138	16.7%
	Polytherapy	102/138	73.9%
Potential drug‐to‐drug interactions	29/138		21%
Therapy changes before starting DAAs	11/29		37.9%
Outcome of the last therapy	Naive	98/138	71%
	Nonresponder	18/138	13%
	Partial responder	2/138	1.4%
	Relapser	12/138	8.7%
	Discontinued due to AEs	8/138	5.8%
Last therapy	Interferon‐based therapy	38/40	95%
	DAAs	2/40	5%
Cirrhosis	55/138		39.8%
Child‐Pugh pretreatment	A	52/55	94.5%
	B	3/55	5.5%
MELD pretreatment	8.96 ± 3.35 (6–20)		
Esophageal varices	18/55		32.7%
Previous bleeding	1/55		1.81%
Previous ascites	7/55		12.7%
Previous encephalopathy	1/55		1.81%
Previous HCC	9/138		6.5%
Cryoglobulin	Present	37/138	26.8%
	Absent	68/138	49.28%
	Not known	33/138	23.91%

Abbreviations: AEs, adverse events; BMI, body mass index; CIRS, Cumulative Illness Rating Scale; DAAs, directly acting antivirals; HBV, hepatitis B virus; HCC, hepatocellular carcinoma; HCV, hepatitis C virus; IDA, intravenous drug addiction; MELD, model of end stage liver disease.

Fifty‐five patients (40%) were cirrhotic and in almost all cases they had preserved liver function (Child‐Pugh A 94%; medium model of end stage liver disease [MELD] score = 9, range = 6–20; refer to Table 1 for further details). The prevalence of episodes of pretherapy liver decompensation was 16% (1 bleeding, 7 ascites, and 1 encephalopathy). Nine patients were diagnosed with HCC before treatment (6.5%), of which eight were uninodular and one was multinodular, but all were BCLC A and all underwent locoregional procedures (5 resections, 2 radio frequency thermal ablation [RFTA], 1 percutaneous ethanol injection [PEI], and 1 trans arterial chemo‐embolization [TACE]) with a complete response before starting antiviral therapy. The average time between the HCC complete response and the beginning of the antiviral treatment was 15 months ± 10 months (median = 1.2 years, and range from 3 months to 2.5 years). Just one patient started treatment 6 months before the HCC complete response and he was diagnosed with recurrent HCC right at the end of treatment with DAA.

Thirty‐seven patients (26.8%) had mixed cryoglobulinemia. Of these, only seven were symptomatic for neuropathy and purpura.

**FIGURE 1:**
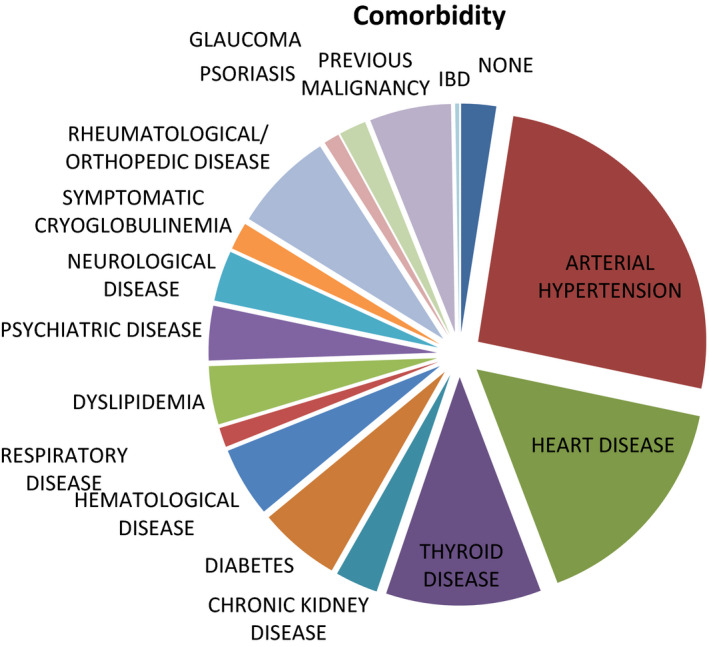
Main comorbidities distribution. IBD, irritable bowel disease

**FIGURE 2:**
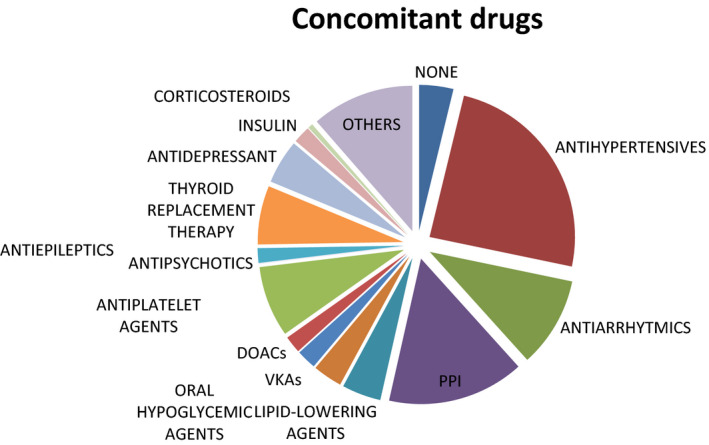
Visual representation of main concomitant drugs. DOAC, direct oral anticoagulant; PPI, proton pump inhibitor; VKA, vitamin K antagonist

Only 7% of patients did not present comorbidities. The most frequent comorbidities were arterial hypertension (68%), heart disease (34%, including chronic heart failure, arrhythmias, ischemic heart disease), thyroid disease (29%, mostly chronic thyroiditis), and diabetes mellitus (15%; Figure 1). One hundred two patients (74%) were exposed to a chronic polytherapy consisting mainly of antihypertensives (65%), proton pump inhibitors (PPIs; 41%), antiplatelet agents (20%), and thyroid hormones (17%; see Figure 2 for more details).

The CIRS severity scale was rated as moderate to severe in most patients (CIRS 3–4 in 65%) for one or two major comorbidities rather than multiple severe comorbidities (refer to Table 1).

### 3.2 Safety and efficacy of treatment

The treatment schedules as well as the data relative to the safety and efficacy of treatment are summarized in Table 2. Potential interactions between the antiviral therapy and patients’ drug therapy were observed in 29 patients (21%) but only in 11 subjects was it necessary to change the home therapy to avoid potentially serious interactions. The most used DAA regimes were Ledipasvir/Sofosbuvir (17%), Glecaprevir/Pibrentasvir (24%), and Grazoprevir/Elbasvir (25%). In terms of length of treatment, most patients (65%) followed a 12‐week treatment, followed by an 8‐week treatment (21%). A minority of patients followed a 16‐week treatment (3%) or a 24‐week therapy (11%).

**TABLE 2:** Treatment schedule and safety and efficacy of treatment in patients

Treatment schedule with DAA	SOF + riba	6/138	4.3%
	SOF/DAC	4/138	2.9%
	SOF/SIM	8/138	5.8%
	SOF/DAC + riba	2/138	1.4%
	LED/SOF	24/138	17.4%
	LED/SOF + riba	2/138	1.4%
	SOF/VEL	20/138	14.5%
	GLE/PIB	34/138	24.6%
	GRA/ELB	35/138	25.3%
	VIEK/EXV	1/138	0.7%
	SOF/VEL/VOX	2/138	1.4%
Length of treatment	8 wk	29/138	21%
	12 wk	90/138	65.2%
	16 wk	4/138	2.9%
	24 wk	15/138	10.9%
AEs due to drug‐to‐drug interaction	2/138		1.45%
SAEs due to drug‐to‐drug interaction	1/2		50%
Adherence to therapy	138/138		100%
AEs	Single	51/138	36.9%
	Multiples	21/138	15.2%
Kind of AEs	Headache	10/138	7.2%
	Nausea/loss of appetite	15/138	10.8%
	Asthenia	22/138	15.9%
	Itch	12/138	8.7%
	Insomnia	8/138	5.8%
	Anemia	2/138	1.4%
Early interruption due to AEs	1/138		0.72%

Abbreviations: AE, adverse event; DAAs, directly acting antivirals; GLE/PIB, glecaprevir/pibrentasvir; GRA/ELB, grazoprevir/elbasvir; LED/SOF, ledipasvir/sofosbuvir; riba, ribavirin; SAE, stand for serious adverse events.; serious adverse event; SOF, sofosbuvir; SOF/DAC, sofosbuvir/daclatasvir; SOF/SIM, sofosbuvir/simeprevir; SOF/VEL, sofosbuvir/velpatasvir; SOF/VEL/VOX, sofosbuvir/velpatasvir/voxilaprevir; VIEK/EXV, viekirax/exviera.

In two patients, we observed an adverse event (AE) due to drug‐to‐drug interaction (1.4%). In both cases, it was caused by an interaction with direct oral anticoagulants (DOACs). One patient developed spontaneous skin ecchymosis without other serious consequences. The other one experienced diverticular bleeding with hospitalization and consequent early stop of the antiviral therapy after 1 month from the start. The patient died 2 years later of heart failure without ever repeating HCV RNA.

There were no other serious adverse events (SAEs). One minor side effect occurred in 37% of patients, of which the most frequently reported were nausea/loss of appetite (11%) and asthenia (16%).

When we correlated each single side effect with each single type of DAA regime in univariate analysis (chi‐square test), we found significant differences in the incidence of itching in patients treated with glecaprevir (GLE)/ pibrentasvir (PIB; 17% vs 3% in other regimens, *P* = 0.02), of insomnia in patients treated with regimens containing sofosbuvir (SOF) compared with regimens not containing it (1.17% vs 0%, *P* = 0.003), and of anemia in patients treated with regimens containing ribavirin compared with regimens without it (2.2% vs 0%, *P* = 0.000). When we performed a logistic regression analysis, limited by the small number of AEs, the GLE/PIB therapy was confirmed to be a risk factor for itching (odds ratio [OR] = 3.6, *P* = 0.03), sofosbuvir‐containing regimens for insomnia (OR = +1000, *P* = 0.005) and treatments containing ribavirin for anemia (OR = +10,000, *P* = 0.007; refer to Table 3 for details). The baseline presence of cirrhosis or elevated CIRS appeared to be protective for the onset of nausea/loss of appetite (Table 3).

**TABLE 3:** Logistic regression correlating the adverse effects with the DAA regimes

	AEs (total)		Headache		Nausea/loss of appetite		Asthenia		Itch		Insomnia		Anemia	
	OR (95% CL)	*P* value	OR (95% CL)	*P* value	OR (95% CL)	*P* value	OR (95% CL)	*P* value	OR (95% CL)	*P* value	OR (95% CL)	*P* value	OR (95% CL)	*P* value
SOF/LED	0.7 (0.2 to 1.7)	0.460	3.2 (0.8 to 12)	0.100	0.3 (0.03 to 2.4)	0.190	**0.17 (0.02 to 1.3)**	**0.030**	—	—	1.4 (0.2 −7.7)	0.65	4.4 (0.2 to 73)	0.310
SOF/VEL	0.9 (0.3 to 2.4)	0.840	1.5 (0.3 to 7.7)	0.620	2.6 (0.7 to 9.4)	0.150	1.3 (0.4 to 4.6)	0.600	—	—	0.8 (0.09 to 7.1)	0.86	—	—
GLE/PIB	1.5 (0.7 to 3.5)	0.250	0.7 (0.1 to 3.8)	0.750	0.8 (0.2 to 3.2)	0.810	1.6 (0.5 to 4.3)	0.350	**3.6 (1 to 12)**	**0.030**	—	—	—	—
GRA/ELB	0.7 (0.3 to 1.6)	0.420	0.7 (0.1 to 3.5)	0.670	1.7 (0.5 to 5.5)	0.360	0.8 (0.2 to 2.4)	0.750	0.9 (0.2 to 3.8)	0.970	—	—	—	—
Treatment associated with ribavirin	2.2 (0.5 to 8.8)	0.240	—	—	1.1 (0.1 to 9.6)	0.900	1.5 (0.3 to 8)	0.610	3.4 (0.6 to 18)	0.190	2.1 (0.2 to 19)	0.52	+ 1000 (0 to +1000)	0.0007
Sofosbuvir‐based therapies	0.9 (0.4 to 1.9)	0.960	1.5 (0.4 to 5.9)	0.480	0.7 (0.2 to 2.2)	0.610	0.8 (0.3 to 2)	0.690	**0.3 (0.08 to 1.2)**	**0.070**	**+ 1000 (0 to +1000)**	**0.005**	**+1000 (0 to +1000)**	**0.090**
CKD	0.7 (0.2 to 2.5)	0.620	1 (0.1 to 9.2)	0.940	—	—	0.9 (0.1 to 4.6)	0.950	2 (0.4 to 10)	0.400	1.4 (0.15 to 12)	0.76	—	—
Cirrhosis	1 (0.8 to 1.3)	0.630	0.4 (0.1 to 1.6)	0.210	**0.19 (0.05 to 0.7)**	**0.007**	**0.2 (0.1 to 0.7)**	**0.007**	0.6 (0.1 to 2)	0.450	0.6 (0.1 to 2.6)	0.54	1000 (0 to 1000)	0.160
CIRS 3–4	0.84 (0.4 to 1.7)	0.640	1.24 (0.3 to 5.1)	0.730	**0.25 (0.08 to 0.8)**	**0.010**	0.9 (0.3 to 2.3)	0.860	1.07 (0.3 to 3.7)	0.910	1.6 (0.3 to 8.4)	0.53	1000 (0 to 1000)	0.180
Short therapy	1.2 (0.5 to 2.9)	0.580	0.9 (0.1 to 4.6)	0.930	1 (0.2 to 3.9)	0.960	1.5 (0.5 to 4.3)	0.440	1.2 (0.3 to 5)	0.720	—	—	—	—
Multiple concomitant drugs	0.8 (0.3 to 1.9)	0.720	0.8 (0.1 to 4)	0.800	0.5 (0.1 to 2.4)	0.380	0.9 (0.3 to 2.8)	0.950	1.7 (0.4 to 6.2)	0.400	—	—	—	—

Abbreviations: AEs, adverse events; CI, confidence interval; CIRS, cumulative illness rating; CKD, chronic kidney disease; GLE/PIB, glecaprevir/pibrentasvir; GRA/ELB, grazoprevir/elbasvir; OR, odds ratio; SOF/LED, sofosbuvir/ledipasvir; SOF/VEL, sofosbuvir/velpatasvir.

The Bold values indicates the statistically significance.

**FIGURE 3:**
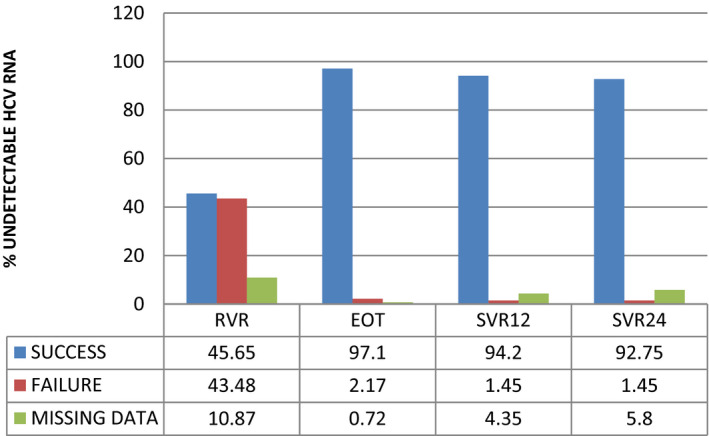
Efficacy of treatment. EOT, end of treatment; HCV, hepatitis C virus; RVR, rapid virological response; SVR, sustained virological response

The efficacy of therapy, evaluated both with the intention‐to‐treat and for per‐protocol analysis, was excellent with an end of treatment (EOT) response in 98% of patients and a rate of sustained virological response (SVR) 12 and SVR 24 of 98% (refer to Figure 3 for details).

### 3.3 Post‐therapy outcome

A total of 12 patients (8.7%) died during follow‐up, nine of which were cirrhotic (Table 4). The causes of death were HCC (4 patients), non‐hepato‐related causes (6 patients who all died of cardiac diseases), and terminal hepatic failure (2 patients). Concerning the last two patients, both scored a Child‐Pugh class A and MELD 10 prior to treatment and did not manifest any previous episodes of decompensation. One patient was previously treated for HCC with a complete response before treatment and no relapse; decompensation with ascites occurred 2 years following the EOT. The other patient had a dysplastic nodule diagnosed as HCC following treatment. The progression of HCC lead to decompensation 1 year later. An average of 479 ± 355 days (median = 381, range = 71–1213) elapsed between the end of therapy and death (refer to Table 4).

**TABLE 4:** Post‐therapy outcome

	Intention‐to‐treat		Per‐protocol
Response to treatment	RVR	63/138 (45.62%)	63/122 (50.81%)
	EOT	134/138(97%)	134/137 (97.8%)
	SVR	12 130/138 (94.2%)	130/132 (98.4%)
	SVR	24 128/138 (92.7%)	128/130 (98.4%)
Post‐treatment ascites	3/55		5.56%
Post‐treatment encephalopathy	1/55		1.81%
HCC post‐treatment	11/138		7.97%
Child‐Pugh post‐treatment	A	49/54	90.7%
	B	4/54	7.4%
	C	1/54	1.8%
MELD post‐treatment	9.6 ± 4.3 (6–25)		
CIRS post‐treatment severity scale	2	63/135	46.6%
	3	26/135	19.2%
	4	46/135	34%
Comorbidity index	0	61/135	45.2%
	1	53/135	39.2%
	2	15/135	11.1%
	3	3/135	2.2%
	4	1/135	0.7%
	5	2/135	1.5%
Deceased	12/138		8.7%
Cause of death	Liver failure	2/12	17%
	HCC	4/12	33%
	Causes unrelated to liver	6/12	50%
Days between the end of therapy and death	479.4 ± 355.73 (71–1213)		

Abbreviations: CIRS, cumulative illness rating; EOT, end of treatment; HCC, hepatocellular carcinoma; MELD, model of end stage liver disease; RVR, rapid virological response; SVR, sustained virological response.

In patients with cirrhosis, the incidence of post‐treatment decompensation episodes was in the order of 7%, with three patients who developed ascites and one patient who experienced an episode of hepatic encephalopathy. The aforementioned episodes happened between the EOT and 12 months following treatment in patients who presented a worsening of Child‐Pugh due to the development of HCC. The incidence of HCC post‐treatment was 8%. Child‐Pugh post‐treatment class A was expressed in 90.7% of patients, with no significant differences compared to pretherapy. More specifically, out of 54 patients scoring Child‐Pugh post‐treatment class A, 43 patients remained in the same class and were rated with the same score as before treatment, two patients remained in class A but improved their score from 6 to 5, three patients remained in class A but worsened their score from 5 to 6, two patients improved their score from B 7–8 to A6, and four patients worsened their score from A 5–6 to B 7–8 or C 10 (all of them presented HCC). MELD had a slight worsening following therapy of about half a point (9.6 post‐therapy compared to 9 pretherapy). CIRS post‐therapy was moderate to severe in 53% of patients (severity scale 3–4) with a slight but statistically significant improvement compared to pretherapy (53.2% vs 65.1%, *P* < 0.000; refer to Table 4 for details).

We compared the blood chemistry tests before and after treatment and found a statistically significant improvement in the value of albumin, total and direct bilirubin, ALT, and AST (Table 5). A slight reduction in alpha‐fetoprotein levels was observed in 77% of patients with a medium decrease of −0.18 × upper limit of normal (ULN).

**TABLE 5:** Blood chemistry trend

Parameters	Before treatment			After treatment			
	Mean	SD	Range	Mean	SD	Range	*P* value
AFP, xUNL	1.19	2.5	0.67–1.73	1.04	3.3	0.35–1.73	0.720
Albumin, g/dL	4.09	0.4	4.04–4.17	4.2	0.35	4.14–4.27	**0.001**
INR	1.18	0.59	1.07–1.28	1.17	0.42	1.09–1.24	0.740
Total bilirubin, mg/dL	0.78	0.33	0.72–0.84	0.73	0.36	0.67–0.79	**0.030**
Direct bilirubin, mg/dL	0.29	0.17	0.26–0.32	0.25	0.14	0.23–0.28	**0.010**
EGFR, with MDRD	77.31	22.89	73–81	78.77	23.86	74–83	0.250
ALT, xULN	1.81	3.5	1.21–2.46	0.85	3.2	0.3–1.4	**0.010**
AST, ULN	1.46	1.32	1.23–1.69	0.55	0.31	0.48–0.60	**0.000**
Hemoglobin, g/dL	13.6	1.5	13.4–13.9	13.6	1.5	13.3–13.9	0.740
White blood cells, cell/mcL	5824	2540	5384–6265	6150	4002	5455–6844	0.130
Neutrophils, cell/mcL	3122	1299	2897–3058	3255	1133	3058–3451	0.190
Platelets, x1000	175.9	71.4	163–188	185.7	85.9	170–200	0.160

Abbreviations: AFP, alpha Fetoprotein; ALT, alanine transaminase; AST, aspartate aminotransferase; EGFR, estimated glomerular filtration rate; INR, international normalized ratio; MDRD, modification of diet in renal disease; xULN, times upper limit of normal.

The Bold values indicates the statistically significance.

It is worth underlining that the majority of patients presented normal alpha‐fetoprotein levels before and after therapy. The lowering was observed both in cirrhotic and noncirrhotic patients, with a normalization expressed in 17.6% of patients. Even patients who developed HCC post‐treatment did not have significant variations with only two patients showing a small increase in alpha‐fetoprotein levels, but always within the normal limit, and with just one patient who presented normal values before therapy and then manifested a large increase in alpha‐fetoprotein levels following therapy (>30 × ULN).

Out of 37 patients who had positive cryoglobulinemia, 16 patients (43%) became negative and 9 (24%) remained positive. Data were missing for the remaining 12 patients (33%). Persistent patients were all cirrhotic, with genotypes 1Bor 2 and a medium cryocrit of 3.18% (minimum 0.8% − maximum 10%). None of them presented hematological or autoimmune comorbidity.

There was also a significant improvement of almost all the ultrasound variables analyzed with a reduction in the size of the liver, an increase in the portal flow rate, a reduction in the longitudinal diameter of the spleen, and, when available, also a significant reduction in the elastographic measurements measured with ARFI or Fibroscan (Table 6).

**TABLE 6:** Ultrasound variables trend

Parameters	Before therapy	After therapy	*P* value (< 0.05)
Liver, cm	14.49 ± 1.78 (14.1–14.8)	14.08 ± 1.66 (13.7–14.3)	**0.004**
Portal vein, mm	11.16 ± 1.83 (10.8–11.5)	10.89 ± 1.91 (10.5–11.2)	0.100
Portal vein velocity, cm/s	16 ± 3.33 (15.3–16.7)	17.2 ± 3.64 (16.5–18)	**0.003**
Spleen, cm	11.2 ± 2.7 (10.7–11.7)	11 ± 2.6 (10.5–11.5)	**0.010**
ARFI, m/s available for 66 patients	1.86 ± 1.51 (1.5–2.2)	1.35 ± 0.65 (1.2–1.5)	**0.003**
Fibroscan, KPa, available for 39 patients	14 ± 9.67 (10.9–17.1)	9.5 ± 4.55 (8–11)	**0.02**

Abbreviation: ARFI, acoustic radiation force impulse.

The Bold values indicates the statistically significance.

Thirteen hepatic nodules were found following treatment (Table 7). Out of these, 11 were HCC: six HCC post‐treatment were recurrences of a previous HCC, whereas the remaining five were de novo HCC. In general, patients who developed HCC, both de novo and recurrent, presented a mean disease duration of 20.35 ± 7.97 years. All patients who developed HCC were cirrhotic with an average of 247 ± 381 days (median = 166, range = 65–1343 days), which elapsed between the end of therapy with DAA and the diagnosis of HCC. Moreover, we found that recurrent HCC developed in less time (medium = 116 days) than de novo HCC (medium = 405 days). Three patients were diagnosed with HCC before the end of the DAA therapy, two of which had recurrent HCC and one de novo HCC.

**TABLE 7:** Hepatocellular carcinoma

	Pre‐DAAs		Post‐DAAs	
Hepatic lesions in US	14/108 (12.96%)		13/108 (12.03%)	
HCC	9/14 (64.28%)		11/13 (84.62%)	
			Recurrent	6/11 (54.55%)
			De novo	5/11 (45.45%)
Multinodular	1/9 (11.11%)		7/11 (63.64%)	
Maximum diameter, cm	4.72 ± 2.94		2.9 ± 1.7 (*P* = 0.29)	
BCLC	A	9/9 (100%)	A	3/11 (27.27%)
			B	2/11 (18.18%)
			C	6/11 (54.54%)
First treatment	Resection	5/9 (55.56%)	Resection	1/11 (9%)
	RFTA	2/9 (22.22%)	RFTA	1/11 (9%)
	TACE	1/9 (11.11%)	TACE	1/11 (9%)
	PEI	1/9 (11.11%)	SIRT	2/11 (18%)
			Sorafenib	3/11 (27%)
			Somatostatin	1/11 (9%)
			BSC	2/11 (18%)
Response to first treatment	CR	9/9 (100%)	CR	4/11 (36%)
			PR	1/11 (9%)
			NR	6/11 (54.5%)
Relapse after first treatment	3/9 (33.33%)		—	
Second treatment	Resection	2/3 (66.67%)	—	
	PEI	1 /3 (33.33%)		
Response to second treatment	CR	3/3 (100%)	—	
Death for HCC	0/9 (0%)		4/11 (36.3%)	
Alpha‐phetoprotein, xULN	2.65 ± 5.5 (range 0.18–5.5)		2.47 ± 7.3 (range 1.4–6.2) (*P* = 0.93)	

Abbreviations: BCLC, Barcelona clinic liver cancer; BSC, best supporting care; CR, complete response; DAAs, directly acting antivirals; HCC, hepatocellular carcinoma; NR, null response; PEI, percutaneous ethanol injection; PR, partial response; RFTA, radio frequency thermal ablation; SIRT, selective internal radiation therapy; TACE, trans arterial chemo‐embolization; xULN, times upper limit of normal; US, ultrasound.

HCC observed after antiviral therapy was smaller, with an average diameter of 2.9 cm, but was more often multinodular (63%), and in 54% of cases in BCLC C stage with the consequent need of palliative or best‐supporting care (Table 7). Four patients (36%) diagnosed with HCC died from disease progression and the death occurred on average 625 ± 383 days (median = 572, range = 253–1213 days) after the end of DAA therapy and 493 ± 405 days (median = 332, range = 203–1278 days) after the diagnosis of HCC.

We did not find any statistically significant difference in the onset of post‐therapy HCC based on the type of DAA used. The one exception is for sofosbuvir‐based schedules (OR = 5, *P* = 0.04). This is clearly a bias because most patients with HCC were cirrhotic and until recently SOF was the only molecule approved in Italy for therapy in this kind of patient The only risk factor clearly associated with the onset of post‐therapy HCC in our analysis was cirrhosis itself (OR = 8, *P *= 0.05).

Finally, we performed a subanalysis dividing the population of our dataset into patients ≥ 80 years old vs patients < 80 years old. No statistically significant difference emerged both in terms of baseline characteristics and in therapy outcome. At the baseline, the two groups were equal in terms of severity of CIRS score (*P* = 0.35), percentage of patients with cirrhosis (*P* = 0.61), previous episode of decompensation (*P* = 0.62), Child Pugh (*P* = 0.96), and MELD score (*P *= 0.95). A small difference, even if not statistically significant, emerged in home therapy, with patients over 80 years old that take more often a polytherapy (*P *= 0.08). A trend toward significance was also observed in the number of patients undergoing short therapy, and greater in the over 80 year patients (*P *= 0.06). No differences were found in incidence of AE (*P* = 0.60) or in efficacy of antiviral therapy (SVR = 12, *P* = 0.20). The post‐therapy outcomes show a similar incidence of decompensation events (*P* = 0.20), HCC (*P* = 0.75), or death (*P* = 0.59; refer to Table 8 for more details).

**TABLE 8:** Comparison of the baseline characteristics and therapy outcome dividing patients by age (≥ 80 years old vs < 80 years old)

	< 80 years old		≥ 80 years old		*P* value
CIRS moderate to severe before treatment, 3–4	58/94		31/44		0.350
CKD, eGFR ≤ 50 ml/min	7/94		6/44		0.250
Concomitant drugs	None	12/94	None	1/44	0.080
	Monotherapy	17/94	Monotherapy	6/44	
	Polytherapy	64/94	Polytherapy	37/44	
Potential drug‐to‐drug interactions	21/94		8/44		0.550
Cirrhosis	36/94		19/44		0.610
Child‐Pugh before treatment	A	34/36	A	18/19	0.960
	B	2/36	B	1/19	
MELD before treatment	9		8.93		0.950
Pretreatment decompensation events	Bleeding	1/36	Bleeding	0/19	0.570
	Ascites	4/36	Ascites	3/19	0.620
	Encephalopathy	1/36	Encephalopathy	0/19	0.460
HCC pretreatment	4/94		5/44		0.110
Short treatment, 8 wk	15/94		13/44		0.060
AEs due to drug‐to‐drug interaction	2/94		0/44		0.320
AEs	36/94		15/44		0.600
RVR	39/94		23/44		0.390
EOT	87/94		43/44		0.450
SVR 12	84/94		37/44		0.200
SVR 24	80/94		36/44		0.390
CIRS moderate to severe 3–4 after treatment	45/94		19/44		0.890
Child‐Pugh after treatment	A	32/35	A	13/15	0.540
	B	2/35	B	2/15	
	C	1/35	C	0/15	
MELD after treatment	9.8		9.33		0.700
Post‐treatment decompensation events	Bleeding	0/36	Bleeding	0/19	‐
	Ascites	1/36	Ascites	2/19	0.200
	Encephalopathy	0/36	Encephalopathy	1/19	0.150
HCC post‐treatment	7/94		4/44		0.750
Death	7/94		5/44		0.590

Abbreviations: AEs, adverse events; CIRS, Cumulative Illness Rating Scale; CKD, chronic kidney disease; eGFR, estimated glomerular filtration rate; EOT, end of treatment; HCC, hepatocellular carcinoma; MELD, model for end‐stage liver disease; RVR, rapid virological response; SVR, sustained virological response.

## 4 DISCUSSION

From our analysis, it emerges that treatment with DAAs is safe and effective even in elderly patients. Although only 6% of patients did not have comorbidities and only 9% did not take any type of concomitant drug therapy, polycomorbidities, and drug polytherapy were not found to be obstacles to start antiviral treatment. The incidence of side effects also had little effect on the beginning of antiviral treatment. Although largely present (37% of patients experienced at least one), the side effects were mild AEs in all cases (but two), which did not require any type of medical management or suspension of treatment.

The efficacy in terms of SVR was thus equal to that obtained in younger patients. However, our data was affected by the number of deaths and patients lost to follow‐up, as well as by the loss of data due to the retrospective design of the study (SVR 12 = 94% intention‐to‐treat = 98% per‐protocol). Only three patients manifested viremia that was still detectable at the end of therapy, two of which were patients treated with GLE/PIB and who subsequently achieved an SVR at 3 months. This positivity can thus be classified as a laboratory error and the EOT response was virtually 100%. Five patients did not reach SVR 12 or 24. Out of these, one patient was the one who discontinued treatment for an SAE, three patients died before reaching the expected timing for SVR 12/24 and only one patient was truly a relapser at 3 months from the end of therapy (0.87%).

The relapser patient was a 74‐year‐old man, with Child‐Pugh A cirrhosis, naive to previous therapies, with no further comorbidities, and who was not taking any drug therapy at the time. Disadvantageous factors for the good progress of therapy were the presence of liver cirrhosis and a very high viral load at baseline (12,422,792 IU, n.v. < 15), whereas compliance with therapy was verified and the only side effect reported was a mild asthenia.

The patient who was discontinued because of drug interaction with DOAC was a 76‐year‐old man, with Child‐Pugh A cirrhosis, naive to previous therapies, with multiple serious comorbidities (CIRS severity index 4, comorbidity index 5) and chronic polypharmacy. Before starting antiviral therapy, the patient was advised to consult a cardiologist and to switch the oral anticoagulant therapy to subcutaneous heparin, but the patient did not follow the instructions. Following the event and the suspension of the antiviral therapy, the patient was asked for a new determination of HCV RNA, which he did not perform. He died 2 years later of acute and chronic heart failure.

The hepatic outcomes following therapy showed an improvement both in terms of ultrasound findings and in terms of blood tests (refer to Tables 5 and 6). In addition, we observed a very low incidence of decompensation events in post‐therapy patients with cirrhosis (6.25%), especially considering that the risk of decompensation for patients with over 20 years of illness is normally in the order of 66% every year. Another factor to consider is that these decompensation events in our series were almost entirely attributable to patients with hepatocarcinoma.

The risk of post‐therapy HCC was 8%, which was higher compared to pretherapy, but was still globally low compared to the quota (22%) reported in previous studies concerning the prevalence of HCC in elderly patients without SVR.^7^, ^8^, ^9^, ^10^ Likewise, all‐cause mortality was in the order of 8% in our dataset compared to 26%, as previously reported, at 10 years without SVR.^7^, ^8^, ^9^, ^10^

When considering extrahepatic outcomes, we found an improvement in the CIRS score, which was mainly driven by noncirrhotic patients (F0–F2 s Metavir) whose comorbidity score increased due to chronic liver disease, a factor that disappeared following treatment with the consequent decrease in the score itself. The aforementioned patients represented 40% of our cohort and were all able to end follow‐up, due to the absence of other hepatic comorbidities.

The same results were confirmed even when a subanalysis was conducted dividing the patients by age (patients ≥ 80 years old vs < 80 years old). No significant differences in the basal characteristics of comorbidities and pharmacotherapy, in the incidence of side effects, and in the response to treatment were observed (Table 8). Furthermore, there was no higher incidence of decompensation events in patients aged over 80 years old compared with younger patients following treatment, although the overall mortality was higher (but not significantly higher) in this cohort of patients.

## 5 CONCLUSIONS

The benefit of DAA therapy in elderly patients was found to mainly concern liver disease and is strongly indicated in patients with cirrhosis, regardless of age. Concerning noncirrhotic patients, the therapy did not appear to affect extrahepatic comorbidities but allowed to end follow‐up in 40% of our patients with consequent savings in terms of resources. It also played an important role in reducing the risk of hepatocellular cancer and all‐cause mortality, if compared with untreated patients. Age should not be an a priori exclusion factor if the patient has a good performance status, even when age is very advanced. There was no difference in efficacy and safety in the treatment with antivirals in patients aged more or less 80 years old.

The large sample size and the long post‐therapy follow‐up enabled us to collect and analyze a lot of data, although the data collected were heterogeneous because of the retrospective design with the consequent difficulty in finding the clinical documentation if not adequately collected, which can be considered the main drawback of our study.

Areas of interest to be explored remain the evaluation of the impact of antiviral therapy on the quality of life and the execution of a cost‐utility analysis, which would be crucial for the correct allocation of health resources.

## CONFLICT OF INTEREST

The authors have no conflict of interest to declare.

## AUTHOR CONTRIBUTIONS

Conception of the work: De Santis and Maggi. Data collection: Lubrano Lobianco. Data analysis and interpretation: Maggi and Lubrano Lobianco. Drafting the article: Maggi. Critical revision of the article: De Santis. Final approval of the version to be published: De Santis, Maggi, and Lubrano Lobianco.
